# Progress in Medicine

**Published:** 1897-02-06

**Authors:** 


### Progress in Medicine.

### RENAL MEDICINE.

A remarkable case of icteric hæmoglobinuria recurring at intervals, in a woman aged twenty-nine, is reported by G. Washington Isaac.[1] There was a history of previous residence in an ague district, but the patient had never suffered from it. The jaundice on each occasion was marked, and lasted several days. A temperature of 104 deg. was noted during one of the attacks, which did not follow any over-exertion. The urine was dark, and contained no blood corpuscles or bile when tested, but amorphous *débris* and hyaline casts. It also showed the absorption bands of oxy-hæmoglobin. This dark urine was passed first in the morning, and afterwards it quickly became normal. Nestor Tirard's[2] remarks on indigestion as a symptom of Bright's disease are too important to be passed over. He lays stress on the extreme caution required if we would exclude Bright's disease when vomiting or diarrhœa is present together with albumen. The vomiting may occur in the early morning or at other times, and is most intractable. Similar resistance to treatment is marked also in the flatulent dyspepsia which is seen in the early stages of the disease, and is often the first symptom that anything is wrong, though frequently misinterpreted. Indigestion is in itself often the cause of kidney disease, and particularly so when it is associated with gout or alcohol or uric acid gravel. We are apt to attach too much importance to the absence of albumen as removing a case from the category of Bright's disease. In advanced cases, the heart, the pulse, or retinal changes may assure us of its presence, though albumen be for a while absent. At other times, the dyspeptic symptoms, vomiting or diarrhœa, may be the sole precursors of the disease. Treatment by way of free purgation, wet packs, and so on, will have more avail than that for the relief of the stomach, though we must take care to regulate the diet to prevent future strain on the kidneys.

Chronic interstitial nephritis is not common in children. Indeed, the small red kidney is often stated to occur almost exclusively in persons over forty; but Leonard Guthrie[3] holds, with Saundby and others, that it is merely the last stage of the acute disease, and he records seven cases verified by post-mortems in children under 15. Besides progressive emaciation and pigmentation, he notices the cardiac hypertrophy and tension with polyuria, vomiting, and gastro-intestinal symptoms, and considers the disease may arise from an acute interstitial nephritis, scarlatina, the irritation of uric acid and oxalates, obstruction of the ureters, or parenchymatous disease. Treatment should be guided especially by the heart and pulse. Thus we should relieve high tension, and where, on the other hand, there was a feeble heart and pulse, digitalis and similar drugs would be useful. Savill remarked on the importance of this paper as showing that this type of Bright's disease was a renal affection, and not a general fibrosis. Indeed, he believes that cardio-vascular hypertrophy may exist as a distinct affection without renal disease. In a lecture by T. H. Green[4] on chronic Bright's disease, the author lays special stress on the remedial conservative action of cardiac hypertrophy and high tension by which a greater amount of blood is made to circulate through the inefficient kidney in a given time, and thus some compensation is effected. Still, when heart failure renders digitalis necessary in these cases, he recommends the addition of nitro-glycerine to obviate the constriction of the vessels, a rule which seems hardly agreeable to what he has said in praise of high tension—though it is no doubt a useful caution. Renal tuberculosis sometimes occurs as a complication of phthisis, as in cases recently recorded by H. M. King, when it may produce considerable pyrexia and rapid death; or it may appear alone, when early recognition is important, since treatment of the general health at this stage may obviate the need for surgical assistance later on. The symptoms, according to F. Tilden Brown,[5] may include pain or tenderness, often the presence of a tumour, œdema, albuminuria, with blood and pus in acid urine, dysuria, pyrexia, turbid urine seen by the cystoscope flowing from one ureter, and the presence of tubercle bacilli. The latter may be confounded with the smegma bacilli, unless thoroughly soaked in alcohol. This difficulty was fully discussed by Goethe in the *Fortschritte der Medicin* of last year. He there pointed out that Lustgarten, Giacomo, and the ordinary carbol-fuchsin with nitric acid stains coloured equally the two bacilli, but if we wash those stained by the latter method in alcohol for ten minutes, the smegma bacilli are decolorised, and the tubercle not. Weichselbaum attains the same end by staining with carbol fuchsin, washing, and then counterstaining with concentrated alcoholic methylene blue. By this plan the smegma and all bacilli except tubercular are stained blue, since the alcohol, if used for more than five minutes, removes the fuchsin from them. Several other discussions of renal tuberculosis are referred to by Bradford.[6] Thus on the question whether it is ascending or descending in its origin he shows that Borrel produced it by injections into the blood stream, and Hamil, having analysed fifty-five cases, found that the bladder and ureters were unaffected in eighteen out of thirty-six instances. The disease occurred twice as often in boys as in girls, and rather more frequently in the right kidney than in the left. The earliest symptoms are incontinence and urinary irritability, later on come hæmaturia and pain, the latter being sometimes paroxysmal like colic. Pyrexia and emaciation appear afterwards. Nephritis in secondary syphilis has been much doubted, but Doederlein[7] reports the case of a youth where the kidneys showed interstitial nephritis, and in other organs there were similar changes. Thus in the lungs and lymphatic glands there was evidence of induration The changes were probably due to an interstitial overgrowth produced by a toxin. There was little albumen, and few casts in the urine. Similar changes were found by Wagner. Furbringer[8] believes that mercurial albuminuria depends much on individual peculiarities, and that care should be taken in administering the drug to patients who have had scarlatina or nephritis, for kidney disease may easily be revived by cold, or the influence of mercury.

The production of localised uræmic symptoms is another matter of dispute. The paralyses have frequently no post-mortem changes to account for them. Localised œdema is often invoked as a cause; but Bradford[9] remarks that there are no strong reasons found in support of the theory, as the paralyses often occur in patients who have no trace of œdema elsewhere in the body. Cerebral anæmia and vasomotor spasm is negatived by the apparent absence of any vasomotor mechanism for the cerebral vessels. Changes in the blood supply seem to be effected by alterations in the calibre of vessels in other areas, notably the splanchnic. Thus Leonard Hill failed to obtain any evidence of active constriction in the cerebral vessels. Bradford returns to the view that the action of toxins direct on the nerve elements is the cause of these paralyses and convulsions. Some of the centres are more irritable than others, and as in ordinary epilepsy the convulsions often begin at one spot and become generalised later on. In the diagnosis of uræmic from other convulsions, Leube only assumes uræmia when other causes can be excluded, and A. R. Edwards[10]

reminds us that difficulties may occur in uræmia itself, for albumen or casts may be absent, and even the amount of area and urine may be normal at the time. Albumen again may appear as a result of nerve lesions, such as cerebral hæmorrhage; even the cardiac symptoms are misleading. Retinitis albuminurica is a valuable symptom, and most frequently in uræmia the pupils are dilated. However, Edwards confesses that the diagnosis between meningitis and uræmia is almost impossible, and that no focal or general sign in the former has failed to appear in uræmia. In the latter, indeed, the paralyses are usually transient. Motor paralyses are rare, the sensorium is more often attacked. Irritation of the motor tracts is common; rarely does it affect the sensorium and cause delirium; never the special senses. Neither the temperature, the breathing, nor the convulsions give any certain indications for diagnosis. Aphasia is occasionally seen, as in Rendu's case,[11] where a man became suddenly unconscious, and on recovery was found to have aphasia, and right brachiplegia with a systolic murmur. A diagnosis of cerebral embolism was made, but later on he was found to be definitely uræmic, and was bled and treated in the hospital, when every symptom disappeared.

Ord[12] describes the causation of hydronephrosis as sometimes congenital, and sometimes dependent on a valvular state or twist of the ureter, sometimes on the presence of tumour, more often through a calculus, and occasionally from a stricture in the urethra or constriction in the prepuce. A curious case resulting from the last-mentioned cause in a boy of eight is reported. In the same paper he bears testimony to the relief he has given by administering dilute nitric acid for renal calculi, though the rationale of it is not clear. Benzoate of ammonia is another drug which he finds very useful for the same purpose. Klemperer[13] has also contributed a paper on the treatment of uric acid gravel, in which he advises the drinking of large quantities of water, the avoidance of saline purgatives, which make the urine concentrated, and of much exercise and sweating. As he adopts the view of its origin from nuclein, he gives only such foods as milk, cheese, bread, and vegetables, which contain but little. Since in many persons this nuclein is excreted as uric acid, though others are able to transform it into urea, similarly he allows only a moderate use of tea and coffee and meat extract which contain xanthin bodies. Finally he gives an alkaline draught every afternoon, and urea in half-ounce doses of a five to ten per cent. aqueous solution to increase the solubility of the uric acid. Friedrich[14] confirms Klemperer's results as to the diuretic action of urea, which appears to stimulate the renal epithelium. In a case of cirrhosis of the liver with healthy kidneys he gave as much as twelve grains thrice in the day, and the urine was increased seven times in quantity.

In the question of a pure milk diet for Bright's disease, recent experiments by Ajello[15] also confirm the accepted view. He found that the albumen was more often increased, the urea, phosphoric anhydride, and sulphur excreted were diminished under a pure milk diet, and the converse under a mixed one. Hence he too insists on the latter diet except in acute cases.

[1] Brit. Med. Jour, Nov. 14. [2] Lancet, Aug. 8. [3] Med. Press, Dec. 2. [4] Clin. Jour., Aug. 26. [5] N.Y Med. Jour., Aug. 15. [6] Practitioner, Sept. [7] B. Med. Jour., Nov. 14. [8] N.YMed. Jour., Aug. 29. [9] Practit., Sept. [10] Am. J. Med. Sc., Aug. [11] Loc. cit. [12] Practition., Nov. [13] B. Med. Jour., Oct., 10. [14] Amer. Med. Surg. Bulletin, Oct. [15] B. Med. Jour., Aug. 29.

